# Idiopathic Osteosclerosis in Orthodontic Patients: A Report of Two Cases

**DOI:** 10.7759/cureus.53426

**Published:** 2024-02-01

**Authors:** Gyanda Mishra, Bernisha R, Seema A Bhogte, Prasad Chitra

**Affiliations:** 1 Orthodontics and Dentofacial Orthopaedics, Army College of Dental Sciences, Secunderabad, IND; 2 Oral Medicine and Radiology, Army College of Dental Sciences, Secunderabad, IND

**Keywords:** radiopacities, orthodontic tooth movement, orthodontics, dense bone islands, idiopathic osteosclerosis

## Abstract

Idiopathic osteosclerosis (IO) is described as a localized radiopacity of unknown etiology. Also known as dense bone islands, enostoses, bone scar, or focal periapical osteopetrosis, it is generally clinically asymptomatic and appears round, elliptical, or irregular in shape on a radiograph. The internal structure is usually homogenous. It should be distinguished from condensing osteitis and other alveolar bone-related radiopacities. This condition may cause changes in tooth position or interfere with orthodontic treatment. Two cases of IO involving the maxilla and mandible are highlighted. Both patients were females and presented with complaints of malocclusion and desired orthodontic correction. One case was detected incidentally on routine radiographic examination of the patient. In contrast, the other case presented as an expansile lesion lingual to the left mandibular first molar and second premolar. Radiographically, both lesions appeared as well-defined radiopaque masses with no surrounding radiolucent rim; the maxillary lesion was irregular in shape, while the mandibular lesion was elliptical. Both patients underwent orthodontic treatment without any adverse sequelae. The clinical and radiographic findings are discussed to facilitate the diagnosis of radiopacities of jaws. Usually asymptomatic and of no clinical significance, IO may occasionally induce root resorption, traumatic occlusion, traumatic/pathologic migration of teeth, and inhibit eruption of teeth. Though orthodontic tooth movement through areas of IO can be undertaken, the rate of tooth movement may be slower due to higher trabecular bone density. Lower force levels are warranted to avoid adverse effects like root resorption and bone hyalinization.

## Introduction

Radiopacities detected incidentally on routine diagnostic radiographs such as orthopantomographs (OPG) present a significant challenge to an orthodontist. The differential diagnosis of radiopaque jaw lesions is extensive and requires consideration of numerous factors, including attenuation pattern, margin characteristics, and relationship to the teeth. Based on the pattern of attenuation, radiopacities can be described as densely sclerotic, ground glass, or mixed lytic-sclerotic, with each category representing a distinct, although occasionally overlapping, differential diagnosis [1]. Dense radiopacities primarily occur due to unerupted teeth, abnormal production of hard tissues related to tooth development, or localized thickening and increased density of the outer layer of bone. By considering the relationship of these densely sclerotic lesions to the adjacent teeth and cortical bone and evaluating the margins of the lesion, it is often possible to arrive at a single diagnosis. On the other hand, lesions with a ground-glass appearance are typically due to disorganized calcification caused by either a neoplastic process or abnormal bone remodeling, as seen in systemic disorders such as renal osteodystrophy. A final diagnosis in such cases requires an assessment of extra gnathic bone findings [1].

Idiopathic osteosclerosis (IO) is a localized radiopaque bony lesion and is of unknown etiology. It is usually asymptomatic, and jaw lesions are typically detected incidentally on approximately 5% (range, 4-31%) of routine dental radiographs [2-6]. Also known as dense bone islands, enostoses, bone scar, or focal periapical osteopetrosis, IO is described as the “internal counterpart” of exostoses with localized hamartomatous growths of cortical bone into the cancellous bone space [2,3,7-10]. IO lesions have been reported not only in the jaws but also in the pelvis, femur, and other long bones [11].

In the jaws, the most common site is the mandibular molar-premolar area, and the size may vary from a few mm to 1-3 cm. The lesions can be round, elliptical, or irregular in shape and may be homogeneously radiopaque or exhibit a heterogeneous appearance. There is a wide variation in the definition of IO, and its biological behavior is not completely understood [3]. Though it is usually asymptomatic, it may cause changes in tooth position or complications during orthodontic treatment [8,10]. The purpose of this paper is to report two cases of IO diagnosed incidentally during the treatment of malocclusion.

## Case presentation

Case report 1

History and Clinical Examination

A 19-year-old female patient reported to the department of orthodontics for correction of malposed teeth. The patient gave a history of extraction of multiple permanent teeth during adolescence due to dental caries. The medical history did not reveal anything of significance. Extraorally, she presented with a class I skeletal pattern, average Frankfort-mandibular plane angle (FMPA), average lower anterior facial height (LAFH), average nasolabial angle, and competent lips with a convex profile. No apparent asymmetry was noted. Intra-oral examination revealed eight missing teeth (16, 12, 26, 36, 37, 45, 46, and 47) and supra-erupted maxillary right and left second premolars and second molars. The molar relationship could not be established, and the extrusion of teeth had led to collapsed interocclusal space in buccal segments bilaterally, mesio-version of left and right lower second molars, and deep bite in the anterior segment.

Radiographic and CBCT Findings

**Figure 1:**
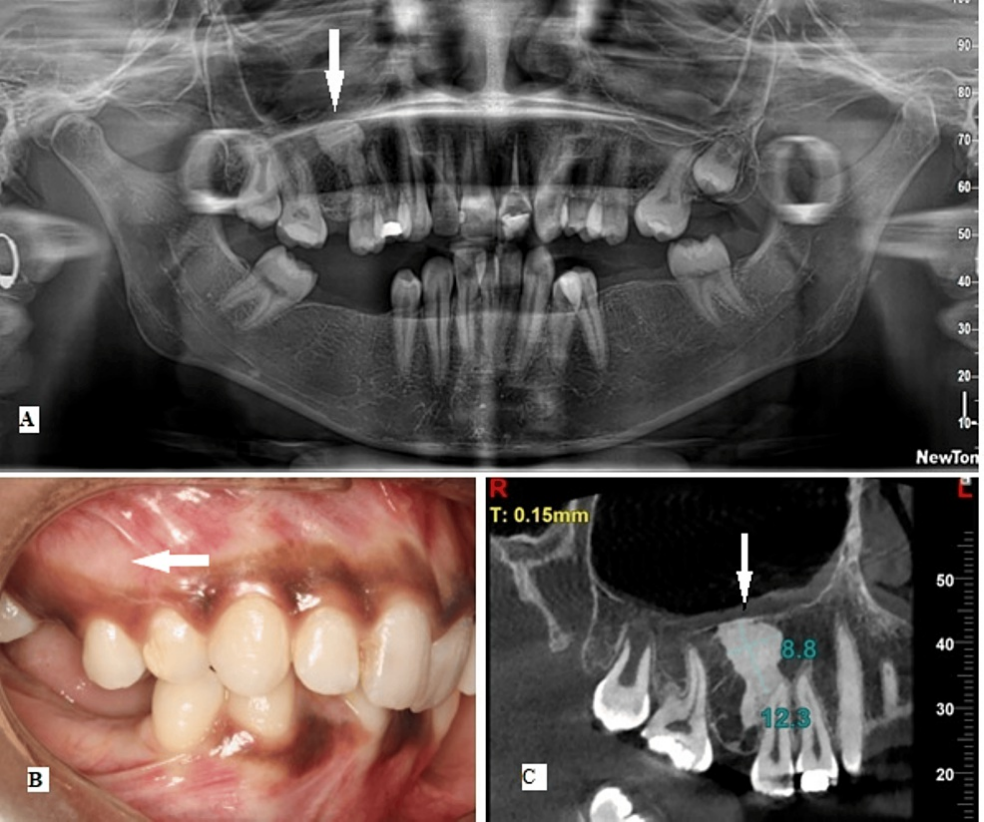
A 19-year-old female presented with multiple missing teeth and malocclusion (A) OPG reveals a solitary round radiopaque mass (arrow), approximately 1 cm in diameter with well-defined margins in the right maxillary second premolar periapical region. (B) The right buccal intra-oral view shows no clinical manifestations (arrow). (C) CBCT image reveals a well-defined irregularly shaped homogeneous radiopacity seen with respect to the periapical region of 15; the lesion was not attached to the tooth and measured approximately 8 × 16 mm in size, extending supero-inferiorly from the floor of the maxillary sinus till the interdental region of 15 and 16. OPG, orthopantomograph

Routine radiographic records, including a lateral cephalogram and OPG, were obtained. The OPG revealed an isolated round mass, approximately 1 cm in diameter, with uniform radiopacity but without a surrounding radiolucent rim in the right maxillary second premolar periapical region (Figure 1A). There were no other associated symptoms, such as swelling or pain (Figure 1B). In order to visualize the alveolar bone quality and quantity, CBCT images of the maxillary right second premolar region were obtained with a NewTom GO Complete Vision machine (Cefla SC, Imola, Italy) with the exposure parameters of 90 peak kilo voltage (kVp), 27.68 milli amperage (mA), 7.47 milligray (mGy) air kerma, and field-of-view of 6 × 6 cm. The views revealed a well-defined irregular radiopacity seen with respect to the periapical region of the right maxillary second premolar. The lesion was not attached to the tooth; it measured approximately 8 × 16 mm in size, extending supero-inferiorly from the floor of the maxillary sinus to the interdental region between the right maxillary second premolar and first molar (Figure 1C).

Differential Diagnosis

Radiographic findings pointed toward a differential diagnosis of IO, condensing osteitis (CO), complex odontoma, periapical osteoma, or ectopic calcification. Normal blood parameters combined with an absence of multiple lesions suggested that there was no systemic disorder affecting calcium metabolism. As the maxillary right second premolar tested positive for vitality and was caries-free, CO was ruled out. The appearance of radiopacity on both the OPG and CBCT views excluded the possibility of any ectopic calcifications. Periapical osteoma was eliminated as the clinical examination did not reveal characteristically painless, smooth nodular swelling. The absence of a radiolucent rim also excluded a diagnosis of mature complex odontoma, mature periapical cemento-osseous dysplasia (PCOD), and mature focal cemento-osseous dysplasia (FCOD).

Provisional Diagnosis

Based on the clinical and radiographic evaluation, a preliminary diagnosis of IO was made. As the lesion was asymptomatic and radiographically benign, it was decided not to confirm the diagnosis with a biopsy. The patient was apprised of possible complications, such as pain, root resorption, and refractoriness to tooth movement.

Orthodontic Management

**Figure 2:**
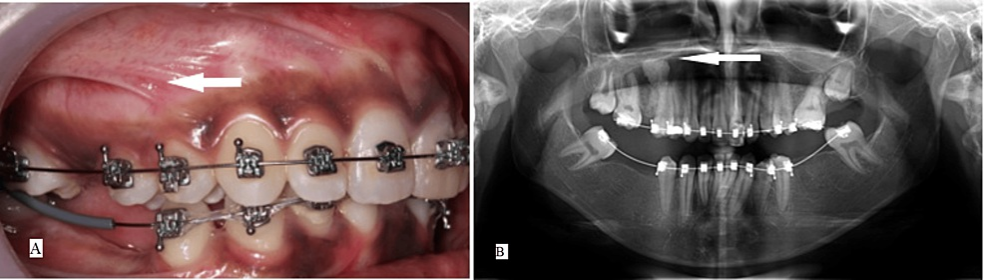
A 19-year-old female presented with multiple missing teeth and malocclusion (A) Intra-oral views 10 months after initiation of fixed orthodontic treatment showing no clinical changes (arrow). (B) Follow-up OPG after 10 months of treatment reveals no changes in the radiographic appearance of the lesion (arrow). OPG, orthopantomograph

After obtaining written consent from the patient, it was decided to proceed with fixed orthodontic treatment with H4 self-ligating appliances (OC orthodontics) to level and align the arches, followed by prosthodontic rehabilitation. Figure 2 shows a 10-month follow-up of the lesion, showing no clinical or radiological changes.

Case report 2

History and Clinical Examination

**Figure 3:**
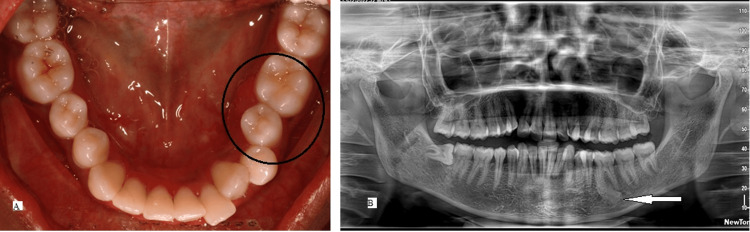
A 21-year-old female presented with malocclusion (A) Intra-oral mandibular arch view shows an expansile lesion lingual to 35 and 36 (black circle) with no remarkable changes in the overlying mucosa. (B) OPG reveals a solitary, unilocular, well-defined radio-dense lesion measuring 7 × 15 mm, located in the posterior mandible and centered at the mesiobuccal root apex of 36 (arrow). OPG, orthopantomograph

A 21-year-old female patient reported to the department of orthodontics with the chief complaint of forwardly placed upper front teeth. Medical and dental history did not reveal anything of significance. The patient had a symmetrical leptoprosopic facial form, skeletal class I relationship, vertical growth pattern, convex profile, and incompetent lips. Intra-oral examination revealed a full complement of maxillary and mandibular teeth with Angle’s class II div 1 subdivision malocclusion. The right mandibular third molar was horizontally impacted and partially erupted. The right maxillary first molar and second premolar were in crossbite relation with moderate crowding in upper and lower anterior segments. A smooth, round mass, approximately 1 cm in diameter, was detected lingual to the left mandibular second premolar and first molar. The lesion extended from the mesial margin of the left mandibular second premolar anteriorly up to the middle of the left first molar posteriorly. The superior margin of the lesion began 5 mm from the lingual marginal gingiva and extended inferiorly toward the lingual vestibule. Upon examination, there was no numbness or tenderness on palpation, and the overlying soft tissues appeared normal. The adjoining teeth were not tender on percussion (Figure 3a).

Radiographic Findings

An OPG showed a solitary, unilocular, well-defined radiopacity measuring 7 × 15 mm, located in the left posterior mandible and centered at the mesiobuccal root apex of the left mandibular first molar (Figure 3b). The lesion demonstrated homogenous radiopacity, was not attached to the root apex, and did not have a radiolucent rim.

Differential Diagnosis

**Figure 4:**
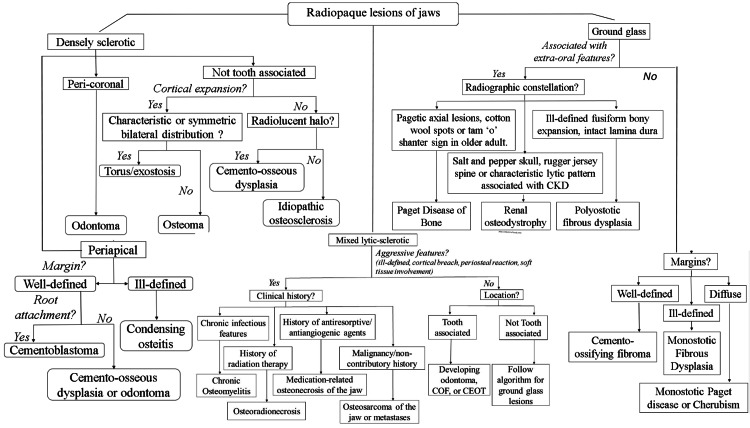
A flowchart depicting the diagnostic approach to radiopaque lesions of the jaws based on clinical history and radiographic characteristics, such as attenuation pattern, location, margins of the lesion, and attachment to roots of associated teeth CEOT, calcifying epithelial odontogenic tumor; COF, cemento-ossifying fibroma; CKD, chronic kidney disease

As the radiopacity was not attached to the roots of the left mandibular first molar, hypercementosis could be ruled out. The lack of a radiolucent rim and the young age of the patient help to refute a diagnosis of mature PCOD and FCOD. Similarly, CO was eliminated as the associated tooth was vital and free from any dental caries or restorations. A subsequent exposure using Clark’s tube shift technique confirmed that the lesion was not a peripheral osteoma. Figure 4 shows a flowchart depicting the diagnostic approach to radiopaque lesions of the jaws.

Definitive Diagnosis

**Figure 5:**
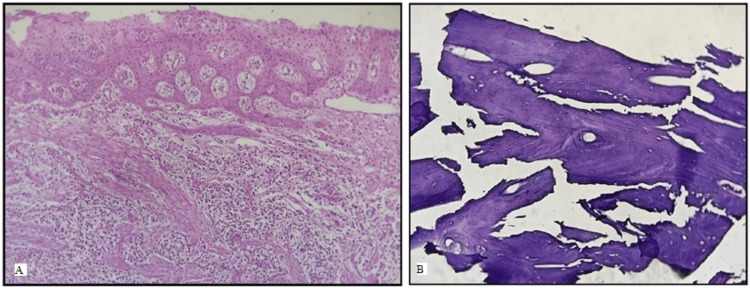
A 21-year-old female presented with malocclusion (A) Histological examination of soft tissue sample at 10× magnification shows stratified squamous epithelium with irregular hyperplasia overlying a densely infiltrated inflamed stroma. (B) The bone tissue examined at 10× magnification shows an irregular haversian system and numerous osteocytic lacunae in concentric lamellae.

Since the lesion presented atypically as a smooth nodular expansile lesion, it was decided to perform a biopsy to confirm the diagnosis with the patient’s consent. After local anesthesia was given, a lingual intra-sulcular incision was made from the left mandibular first molar to the left mandibular first premolar, and a full-thickness triangular flap was elevated. A soft tissue biopsy sample measuring 0.5 × 0.5 cm and a core hard tissue sample measuring 0.5 × 0.5 cm were taken from the dense bone. On histologic examination, the soft tissue showed stratified squamous epithelium with irregular hyperplasia overlying a densely infiltrated inflamed stroma (Figure 5a). The bone tissue showed an irregular haversian system and numerous osteocytic lacunae in concentric lamellae (Figure 5b). The patient remained asymptomatic, and healing was satisfactory without any neurosensory complications. In view of the clinical, radiographic, and biopsy findings, a diagnosis of IO was ascertained.

Orthodontic Management

**Figure 6:**
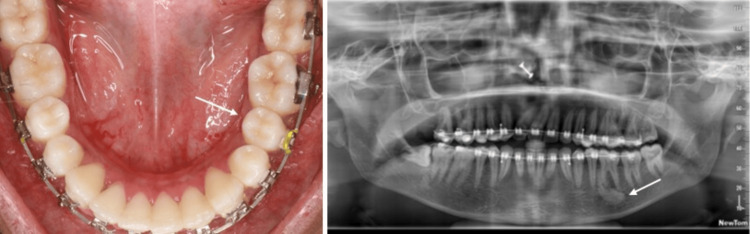
A 21-year-old female presented with malocclusion (A) Intra-oral view shows no change in the clinical appearance of the lesion 11 months after initiation of treatment (arrow). (B) 11-month follow-up OPG reveals no changes in the radiographic appearance of the lesion (arrow). OPG, orthopantomograph

The case was then treated with fixed orthodontic appliances without any extractions; 0.022″ × 0.028″ MBT brackets were bonded, and sequential leveling and alignment were commenced. At the time of writing this case report, the patient had 0.018″ stainless steel wires in both arches. Figure 6 depicts the mid-stage clinical and radiographic views of the case.

## Discussion

Epidemiology, clinical, and radiographic presentation

The reported prevalence rates of IO vary widely between 4% and 31% due to ambiguity in definition and diagnostic criteria [3]. Reports of gender incidence vary. While Geist and Katz, Yonetsu et al., and Kawai et al. found no statistically significant difference between the prevalence of IO in males and females, others have reported IO to be nearly twice as common in females than males [2,4,5,12-14]. It has been reported more often in black female patients [15]. Although it may be seen at any age, the incidence of IO peaks in the third decade of life and declines after the fourth decade [2-5,12]. It is typically asymptomatic and reported as an incidental finding on a radiograph [10]. It is more common in the mandible than the maxilla and is most commonly located in the mandibular molar-premolar region [2-5,10,12]. The usual diagnosis is by radiographic examination and patient history. Biopsy and CT are rarely needed. Geist and Katz have given an extensive description of radiopacities, which could be identified as IO [12]. On radiographs, it appears as a well-defined, round, triangular, oval, or irregular, solitary (but can be multiple) sclerotic lesion with sharp margins without a radiolucent rim. The size may vary from a few mm to 2 cm, and it may be located periapically (80%) or remote to teeth (20%) [3,5,12]. Root resorption and tooth movement are rare. If it blends into bone cortices, it does so with no expansion or thinning. The internal pattern of IO can vary from a ground glass-like pattern to one that is uniformly radiopaque. In some cases, heterogeneous radiolucent areas may be visualized depending on the thickness of the lesion [10]. There is no sign of inflammation of the teeth, and if associated with a root, the periodontal ligament space is preserved.

The etiology of IO is mostly unknown (ergo, the name is idiopathic), and many possible explanations have been proposed for its occurrence. A reactive origin is suggested based on the occurrence of mild inflammation and trauma, with bone deposited in response to unusual occlusal forces [4,12]. Goaz and White affirm that IO might be caused by the resorption of retained deciduous molar roots and its subsequent replacement by sclerotic bone [15]. It has been considered an anatomic variation analogous to tori [16]. Some authors have postulated a developmental etiology, as the lesion is reported mostly during the first three decades of life [16]. Miloglu et al. surmise that these radiopacities might be developmental variations of normal bony architecture unrelated to local stimuli, which could arise at any age and any location in the jaws [13]. It has also been reported that IO may be an earlier CO, where the cause has been eliminated, such as a chronic periapical lesion, periodontal disease, pericoronitis, and occlusal trauma [4]. The current consensus is that IO is a developmental variation of normal bone architecture unrelated to local stimuli [17]. Interestingly, an increasing incidence of IO is associated with colorectal cancer or adenoma [10,14]. Panoramic radiographs have even been described as a marker for the detection of colorectal neoplasia in first‑degree relatives [18]. However, the patients in this case report did not have any history of colorectal cancer/adenoma.

Diagnosis

Differential diagnosis includes CO; mature fibro-osseous lesions of periodontal ligament origin, such as PCOD and FCOD; hypercementosis; exostoses (tori); and abnormally dense alveolar bone induced by heavy occlusal stress [10,15,17]. CO may resemble IO; however, associated teeth are always non-vital in CO. According to Kawai et al., opacifications with thickened trabeculae and narrowed spaces with margins that blend should be called CO, even if they are located under the apices of seemingly sound teeth or teeth with small restorations [4]. On the other hand, when radiopacities present as a homogeneous mass without spaces and with well-contrasted margins, they should be considered as IO, even if they are located subjacent to the apices of deep carious teeth or teeth with inadequate restorations [4].

Benign fibro-osseous lesions, such as fibrous dysplasia, ossifying fibroma, and osseous dysplasia, pose a challenge in terms of distinguishing them based on radiological findings. These lesions can display radiolucency or a combination of radiolucency and radiopacity, or even radiopacity alone, depending on their level of maturity. Conversely, advanced-stage osseous dysplasia can manifest as a uniform radiopaque mass at the root apex of a vital tooth, separated from normal bone by a thin radiolucent halo. Unless the radiopaque area is encompassed by a radiolucent rim, it becomes difficult to differentiate the lesion from IO [15]. 

The compound type of odontomes radiographically exhibits a well-organized multiple malformed tooth or denticle of varying size and shape surrounded by a narrow radiolucent band, while the complex type presents with a large, irregular calcified mass surrounded by a narrow and even radiolucent periphery. Complex odontomas might be difficult to distinguish from IO but are marked by a greater radiodensity and a radiolucent rim corresponding to deposits of enamel and periodontal ligament, respectively. They are also infrequently situated periapically and more often seen over the crown of an unerupted (impacted) tooth or between the roots of teeth [15].

Sclerosis induced by occlusal trauma to an isolated tooth is usually not localized to the periapical region but involves the entire alveolar process around the tooth [15]. Hypercementosis may be differentiated from IO by the club-shaped appearance of the affected tooth and well-defined radiolucent periodontal ligament space delineating the lesion from adjacent normal bone [15].

Some rarities may also be mistaken for IO, such as Paget’s disease, cementoblastoma, osteoblastoma, osteogenic sarcoma, chondrosarcoma, metastatic prostatic carcinoma, and hamartoma [15]. Osteoblastoma and cementoblastoma begin as radiolucent lesions and show progressive radiopacity as the lesion matures. Previous radiographs may help establish the course of the development of the lesion. Metastatic neoplasia may present with a history of treatment for a primary tumor and some clinical symptoms, along with increased levels of serum acid phosphatase. Chondrosarcoma and osteogenic sarcomas may show ragged radiolucent areas within the radiopacity.

Juvenile mandibular chronic osteomyelitis and pseudocysts may also present with radiopacity; nonetheless, these lesions may have pus discharge. The border of lesions is not well-defined, especially in osteomyelitis, and patients may complain of pain.

Clinical significance and management

Areas of IO are not of clinical significance except that they should be differentiated from CO, since teeth associated with CO may require endodontic treatment. No treatment is necessary. Periodic follow-ups should include serial radiographs that show minimal growth or regression. Rarely, if root resorption is observed, the associated tooth may require endodontic treatment or extraction [15].

Orthodontic implications

IO has occasionally been reported to induce root resorption, traumatic occlusion, and traumatic/pathologic migration of teeth and may inhibit the eruption of teeth [3,10,12,19]. Marques Silva et al. suggest that IO may cause changes in tooth position or problems during orthodontic treatment and reported a case of tooth resorption caused by ectopic eruption associated with IO [8].

In the first case in this report, extrusion of the maxillary right second premolar was observed. It is possible that extraction of mandibular molars caused the maxillary right second premolar to supra-erupt due to a lack of opposing occlusal contacts. It may also be possible that the lesion pre-dated the extractions and caused extrusion of the tooth, which would lend credence to the etiological hypothesis based on traumatic occlusion. A diagnosis was made through clinical and radiographic findings by considering and excluding a differential diagnosis of complex odontoma, CO, PCOD, FCOD, periapical osteoma, and ectopic calcification. No radical intervention was carried out as the lesion did not show any signs of malignancy or rapid osteolytic changes. In the second case reported in this paper, although the radiographic appearance was suggestive of IO, the lesion presented clinically as an asymptomatic bony swelling in the left mandibular first molar and second premolar region in contrast to the usual presentation. Thus, a biopsy and histological examination were performed to confirm the diagnosis. Both patients remained asymptomatic through orthodontic treatment.

The bone surrounding IO displays typical structure and function, with the notable characteristic of increased trabecular density. Within this area, teeth can be repositioned, and osseo-integrating implants and mini-implants can be inserted without further consequences, provided there is no infectious component and the fundamental biological principles of orthodontic tooth movement are adequately considered [20]. Orthodontic forces lead to bone deflection, a structural deformation that absorbs a portion of the applied force, subsequently transferring the remaining force to the periodontal ligament. Optimal levels of orthodontic force permit bone remodeling and tooth movement without inducing necrosis of periodontal vessels and subsequent hyalinization. The heightened bone density in the IO region prevents this bone deflection, thereby transferring the entirety of the force to the periodontal ligament. Consequently, normal and conventional forces become detrimental to the periodontal ligament during tooth movement along IO areas. The rate of remodeling or turnover in the IO region is slower, resembling the process that occurs in cortical bone rather than trabecular bone, resulting in a reduced rate of tooth movement. Higher forces also carry the risk of damaging cementoblasts and potentially causing more severe root resorption.

To ensure favorable tooth movement and minimize the possibilities of induced root resorption, light orthodontic forces should be applied. The use of thermally activated nickel-titanium (NiTi) arch wires produces light and continuous forces, even when deflected. This force characteristic is highly desirable in the initial stages of orthodontic treatment, as it promotes tooth movement with minimal risk of root resorption and damage to the supporting tissues while also minimizing patient discomfort. According to the analogy proposed by Consolaro and Consolaro, reducing the force can be likened to a “discount or compensation,” enabling a normal rate of tooth movement despite the absence of bone deflection due to the increased density [20].

## Conclusions

The term IO refers to a localized radiopacity that is not the sequela of infection or systemic disease. IO has no definite etiology, may remain asymptomatic, and without any changes for long periods of time. Routine CT and biopsy investigations are not recommended for these lesions. Orthodontic tooth movement through the IO lesion can be undertaken; however, the rate of tooth movement may be slower due to high trabecular bone density. Orthodontic treatment in areas of IO may warrant lower force levels to avoid adverse effects like root resorption and bone hyalinization. Utilization of NiTi closed coil springs and thermally active NiTi wires, applying light, continuous force should be considered. Periodic follow-up and monitoring are suggested.
